# Pain in chronic liver disease compared to other chronic conditions: Results from a contemporary nationally representative cohort study

**DOI:** 10.1097/HC9.0000000000000605

**Published:** 2024-12-11

**Authors:** Grace Y. Zhang, Aly Cortella, Jennifer C. Lai, Jessica B. Rubin

**Affiliations:** 1 Division of Gastroenterology and Hepatology, Department of Medicine, University of California San Francisco California, USA; 2 Department of Epidemiology and Biostatistics, University of California San Francisco California, USA

**Keywords:** chronic disease, chronic liver disease, opiod, pain, symptom management

## Abstract

**Background:**

Pain is common in patients with chronic liver disease. Our limited understanding of patterns and severity of pain in this population hinders the development of effective cirrhosis-specific pain management strategies.

**Methods:**

Using cross-sectional data from the 2016–2021 National Health Interview Survey, we examined rates, severity, and functional limitations due to pain in respondents with liver disease (viral hepatitis, cirrhosis, and liver cancer), compared to the general population and those with other chronic conditions associated with pain (ie, arthritis, diabetes, and chronic kidney disease). Categorical and continuous variables were compared using χ^2^ and *t* test. Multivariable logistic regression was used to determine the predictors associated with pain and opioid use.

**Results:**

Our liver disease cohort comprised 5267 participants (63% viral hepatitis, 49% cirrhosis, and 2% liver cancer). Participants with liver disease were more likely to report pain than those without liver disease (42% vs. 22%); they were also more likely to report severe pain (42% vs. 30%) and functional limitations by pain (28% vs. 13%) (*p* < 0.001 for all). On multivariable logistic regression, liver disease is an independent predictor of pain (OR: 2.31, 95% CI: 2.05–2.59, *p* < 0.001), even after adjustment for demographic factors. Liver disease respondents had similar rates of pain as those with diabetes (*p* = 0.8) and were more functionally limited by pain than those with arthritis (*p* < 0.001). Adjusted for demographic and pain-related factors, liver disease was also an independent predictor of chronic opioid use (OR: 1.47, 95% CI: 1.12–1.92, *p* = 0.0054).

**Conclusions:**

Liver disease independently increases the likelihood of experiencing widespread and debilitating pain. Clinicians should consider liver disease a painful condition, ensuring that they are frequently assessing and appropriately treating pain in all liver disease patients.

## INTRODUCTION

Pain is a prevalent symptom in patients with chronic liver disease, especially those with cirrhosis. It has been reported that up to 80% of patients with cirrhosis experience chronic pain.^1^,^2^,^3^ Pain can significantly impair quality of life, contributing to symptoms such as sleep disturbances and psychological distress,^1^ many of which are already present in patients with cirrhosis. Managing pain in chronic liver disease is complex due to factors such as HE, impaired drug metabolism, and the potential hepatotoxicity of common analgesics. Health care providers often struggle with how to treat patients with chronic liver disease and cirrhosis, with the risk of potentially undertreating their pain. Our limited understanding of patterns of pain in this population, as well as differences compared to patients with other chronic conditions, hinders the development of effective cirrhosis-specific pain management strategies.

The National Health Interview Survey (NHIS)—an annual survey of ~50,000 representative households in the United States—has been used extensively to monitor incidence and trends in chronic pain.^4^,^5^,^6^ It is thought to be one of the top sources for such studies due to its concise, validated questions that encompass a wide spectrum of pain-related areas.^7^ It has also been used to characterize rates of chronic diseases, including chronic liver disease.^8^ In this study, we used a multi-year, cross-sectional, national cohort of participants from the NHIS to assess and characterize pain and opioid use in participants with and without self-reported liver disease and also to compare pain in those with liver disease to individuals with other chronic conditions.

## METHODS

### Study design and source of data

We performed a retrospective study using cross-sectional data from the NHIS, an annual survey conducted by the CDC and the National Center for Health Statistics, with the goal of describing health status, health care utilization, access to care, and health behaviors of the noninstitutionalized population of the United States. NHIS data are based on personal interviews with ~50,000 unique US households each year. NHIS data are cross-sectional, and households are generally not surveyed across multiple years. The results of the surveys are publicly accessible (https://www.cdc.gov/nchs/nhis/index.htm). NHIS includes the following self-reported demographic variables for most survey years: age, sex, race, ethnicity, employment status, education, insurance status, veteran status, and urban/rural living environment.

### Identifying liver disease and other chronic conditions

We identified participants with self-reported liver disease (ie, viral hepatitis, cirrhosis, or liver cancer) using their responses to the questions in the 2016–2021 NHIS that specifically asked about current or past diagnoses of hepatitis, cirrhosis/liver condition, or liver cancer (Supplemental Table S1, http://links.lww.com/HC9/B796). We excluded data from 2019, given the limited ability to identify participants with liver disease from this year’s surveys. For our primary analyses, we used a composite predictor variable including any of these diagnoses, though we performed subgroup analyses using the 3 diagnoses individually. In addition to comparing those with chronic liver disease to the general population, we also identified patients with diabetes, arthritis, and chronic kidney disease as additional comparator groups. These diseases were selected because of their known association with chronic pain (definitions shown in Supplemental Table S1, http://links.lww.com/HC9/B796).

### Identifying and characterizing pain

All years of the NHIS in this study included questions regarding pain frequency, severity, and functional limitations due to pain over the preceding 3–6 months (Supplemental Table S2, http://links.lww.com/HC9/B796). We compared responses to these questions between respondents with and without liver disease. We created binary definitions of pain and functional limitation outcomes, defined as symptoms on some, most, or all days. We defined severe pain as pain that was reported as “a lot” (highest on a 3-point scale: 1, a little; 2, somewhere in between a little and a lot; 3, a lot).

The 2021 NHIS additionally queried respondents about specific pain locations (eg, back, hands, hips, migraine, and abdominal pain), which was used to compare the location and widespreadness of pain between participants with liver disease and those without liver disease. The 2021 NHIS additionally queried respondents about overall life satisfaction, rated on a scale from 0 (very dissatisfied) to 10 (very satisfied)—this was used as a surrogate for quality of life, which we compared among those with and without pain. The 2020 NHIS queried respondents’ opioid use behaviors over the preceding year, as well as indications (ie, acute vs. chronic) and frequency of opioid use. We used these data to compare patterns of opioid use among participants with and without liver disease.

### Statistical analysis

Categorical and continuous variables were compared using chi-square and *t* tests, respectively. To identify the association between liver disease and our pain-related outcomes, we performed multivariable regression analyses. Simple regressions were performed for the main predictor and all covariates individually. Multivariable models included demographic factors (age, sex, race, ethnicity, education, and urbanicity) that were predicted to be associated with pain or opioid use. Presence, severity of, and functional limitations due to pain were also included in adjusted models for predicting opioid use. Analyses were performed using R (R version 3.6.3) and SAS 9.4. To assess changes in pain reporting over time, we used logistic regression to model the proportion of respondents reporting pain with year as the primary predictor.

## RESULTS

### Liver disease cohort

Our liver disease cohort comprised 5267 participants over the years 2016–2021 (excluding 2019): 63% had a history of viral hepatitis, 49% had cirrhosis, and 2% had liver cancer (patients could have multiple of these conditions). Participants with liver disease had a mean age of 58 (SD: 15) years. Eighty-one percent identified as White and 14% identified as Hispanic, while 51% of our cohort identified as female. Participants with liver disease were largely high-school educated (36%), unemployed (60%), insured (95%), non-Veterans (86%), and living in large central metropolitan areas (27%) (Table 1).

**TABLE 1:** Demographic characteristics among NHIS respondents with self-reported liver disease with and without pain 2016–2021

Demographic characteristics (reported as % unless otherwise specified)	Overall, N = 5267	Pain, n = 2235 (42%)	No pain, n = 3032 (58%)	*p*
Age, mean (SD)	58 (15)	60 (13)	57 (16)	<0.001
Sex				
Female	51	48	54	<0.001
Male	49	52	46	
Race				
Alaskan Indian/Alaska Native	2	2	1	<0.001
Asian	5	2	8	
Black/African American	9	9	8	
White	81	83	80	
Other/Multiple races	2	3	2	
Refused/not ascertained	1	1	1	
Hispanic ethnicity	14	11	14	<0.001
Education^a^				
Up to high school	36	40	33	<0.001
Up to associates	32	37	28	
Bachelor’s degree	18	14	21	
Postgraduate degree	13	8	18	
Refused/nonascertained	1	1	0.4	
Employed	40	28	48	<0.001
Insured^a^	95	96	94	0.90
Veteran^a^	14	14	14	0.16
Urbanicity^a^				
Large central metro	27	22	31	0.14
Large fringe metro	22	21	23	
Medium and small metro	33	38	29	
Nonmetropolitan	18	19	17	

^a^ Lower n due to missing data from several years where certain demographic variables were not captured due to survey variability (total participants reporting liver disease in 2016, n = 1561; 2017, n = 1244; 2018, n = 1178).

Abbreviation: NHIS, National Health Interview Survey.

### Pain among respondents with liver disease

**FIGURE 1:**
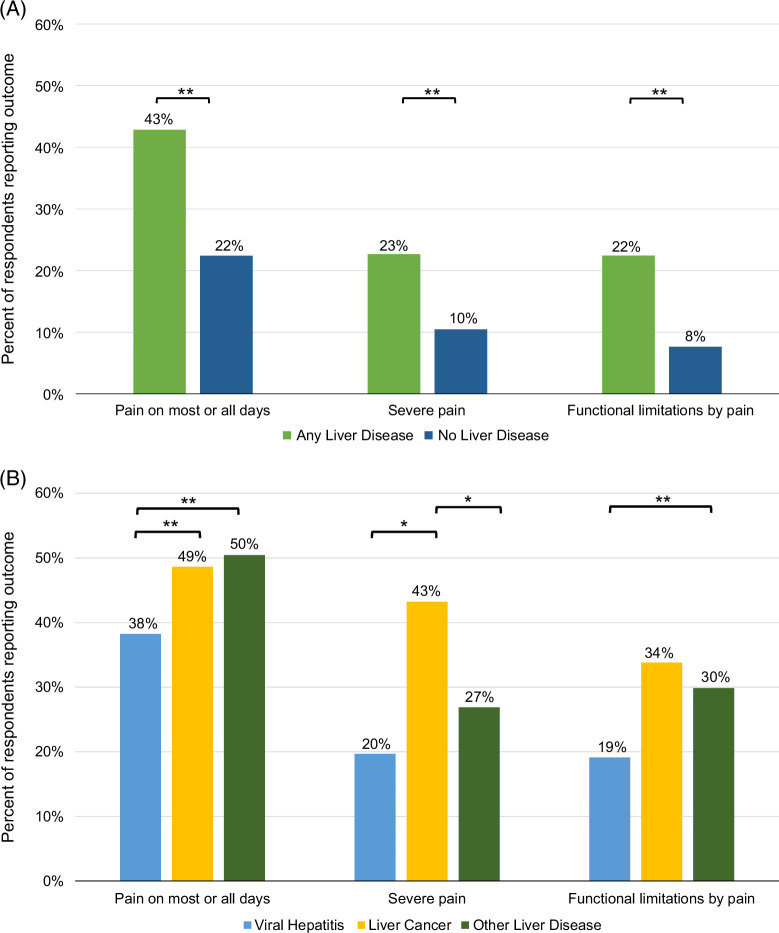
Comparing reports of pain frequency, severity, and functional limitations for (A) participants with liver disease (n = 5267) versus those without liver disease (n = 139,900) and (B) participants with liver disease separated into types of liver disease, including viral, cancer, and other (cirrhosis). **p < 0.001, *p < 0.05.

Compared to participants without liver disease (n = 138,900), participants with liver disease were more likely to report having pain on most or all days (43% vs. 22%, *p* < 0.001). They were also more likely to report severe pain (23% vs. 10%, *p* < 0.001) and functional limitations by pain (22% vs. 8%, *p* < 0.001) (Figure 1A).

Among respondents with liver disease, those with liver cancer or cirrhosis were more likely to report having pain most days or every day compared to those with viral hepatitis only (49% vs. 50% vs. 38% respectively, *p* < 0.001) (Figure 1B). Similarly, on logistic regression adjusting for demographic factors, having viral hepatitis alone was associated with a lower likelihood of pain compared to having cirrhosis or liver cancer (Supplemental Table S4, http://links.lww.com/HC9/B796). Respondents with liver cancer were more likely to report severe pain compared to those with hepatitis or cirrhosis (43% vs. 20% vs. 27%, *p* < 0.05 for both), and were more likely to report functional limitation due to pain though this did not reach statistical significance (34% vs. 19% vs. 30%). Those with cirrhosis were more likely to have functional limitations by pain than those with viral hepatitis (30% vs. 19%, *p* < 0.001).

On the unadjusted logistic regression model, those with liver disease had 2.56 times the odds of reporting pain compared to those without liver disease (OR: 2.56, 95% CI: 2.42–2.70, *p* < 0.001) (Table 2). On multivariable regression, after adjusting for age, sex, race, ethnicity, education, and urbanicity, liver disease remained an independent predictor of pain (OR: 2.31, 95% CI: 2.05–2.59, *p* < 0.001). This remained true in a sensitivity analysis which also adjusted for other painful comorbidities (eg, diabetes, chronic kidney disease, and arthritis) (Supplemental Table S3, http://links.lww.com/HC9/B796).

**TABLE 2:** Unadjusted and adjusted logistic regression predicting pain

Predictors of pain	Unadjusted OR (95% CI)	*p*	Adjusted OR (95% CI)	*p*
Liver disease				
No	Reference		Reference	
Yes	2.56 (2.42, 2.70)	<0.0001	2.31 (2.05, 2.59)	<0.0001
Age	1.29 (1.28, 1.3)	<0.0001	1.02 (1.08, 1.17)	<0.0001
Sex				
Female	1.18 (1.15, 1.21)	<0.0001	1.12 (1.08, 1.17)	<0.0001
Male	Reference		Reference	
Race				
Alaskan/Indian	1.19 (1.05, 1.33)	0.004	1.37 (1.11, 1.68)	0.003
Asian	0.31 (0.29, 0.34)	<0.0001	0.36 (0.32, 0.41)	<0.0001
Black/African	0.85 (0.82, 0.89)	<0.0001	0.90 (0.84, 0.96)	0.001
Other/Multiple	1.19 (1.10, 1.29)	<0.0001	1.33 (1.17, 1.51)	<0.0001
White	Reference		Reference	
Ethnicity				
Non-Hispanic	Reference		Reference	
Hispanic	0.63 (0.60, 0.66)	<0.0001	0.72 (0.67, 0.78)	<0.0001
Education				
Never attended	0.67 (0.36, 1.27)	0.224	0.88 (0.41, 1.91)	0.753
High school	Reference		Reference	
Associates degree	0.97 (0.93, 1.07)	0.206	0.99 (0.94, 1.04)	0.640
Bachelor’s degree	0.54 (0.52, 0.57)	<0.0001	0.61 (0.58, 0.65)	<0.0001
Postgraduate degree	0.52 (0.49, 0.56)	<0.0001	0.56 (0.52, 0.59)	<0.0001
Urbanicity				
Large central metropolitan	Reference		Reference	
Large fringe metropolitan	1.16 (1.10, 1.22)	<0.0001	1.00 (0.95, 1.06)	0.922
Medium and small metropolitan	1.47 (1.40, 1.54)	<0.0001	1.20 (1.14, 1.26)	<0.0001
Nonmetropolitan	1.91 (1.80, 2.02)	<0.0001	1.34 (1.26, 1.43)	<0.0001

**FIGURE 2:**
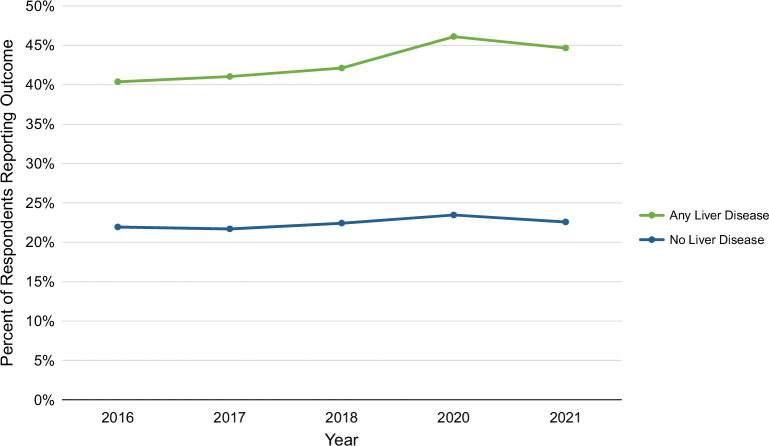
Pain among respondents with and without liver disease by year from 2016 to 2021.

Over the years studied, there was an increase in pain among all respondents—including those with and without liver disease—over time, though rates of pain appear to be rising more rapidly among those with liver disease. Among those with liver disease, for each additional year, there was a 4% increase in the odds of reporting pain, while among those without liver disease, for each additional year, there was a 1.5% increase in the odds of reporting pain (*p* < 0.0001 for both) (Figure 2).

**FIGURE 3:**
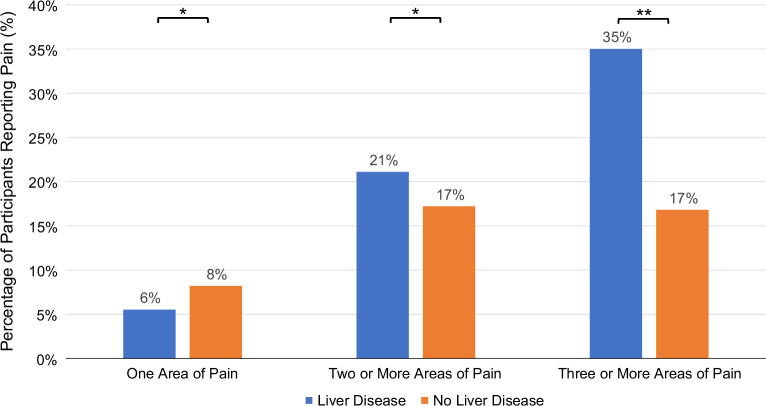
Percentage of participants with and without liver disease reporting pain in 1, 2 or more, and 3 or more locations. Locations interrogated include abdominal pain, migraine, back pain, hand pain, jaw pain, and hip pain. **p < 0.001, *p < 0.05.

Using 2021 survey data, we evaluated the location of reported pain (abdominal pain, migraines, back pain, hand pain, tooth pain, and hip pain) among those with and without liver disease. While the distribution of pain locations was similar between participants with and without liver disease, participants with liver disease were more likely to report pain in multiple locations compared to participants without liver disease (pain in 3 areas: 35% vs. 17%, *p* < 0.001) (Figure 3).

### Pain in liver disease compared with other chronic conditions

**FIGURE 4:**
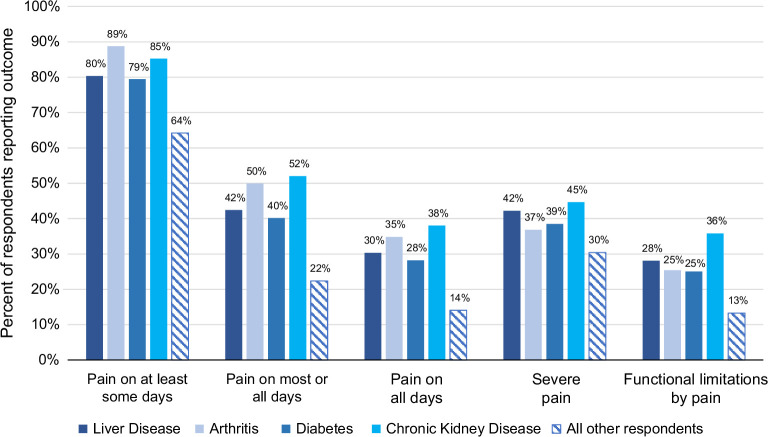
Comparing reports of pain frequency, severity, and functional limitations for liver disease and other chronic conditions, including arthritis, chronic kidney disease, diabetes mellitus, and the general population without liver disease.

NHIS respondents with liver disease were less likely to report pain compared to those with chronic kidney disease or arthritis (42% vs. 52% vs. 50%, *p* < 0.001 for both) but had similar rates of pain as those with diabetes (40%, *p* = 0.8) (Figure 4). The proportion of respondents with severe pain was similar between those with liver disease and those with 1 of the other 3 chronic conditions. Liver disease participants were more likely to be functionally limited by pain than those with arthritis (*p* < 0.001).

### Association between pain and life satisfaction

**FIGURE 5:**
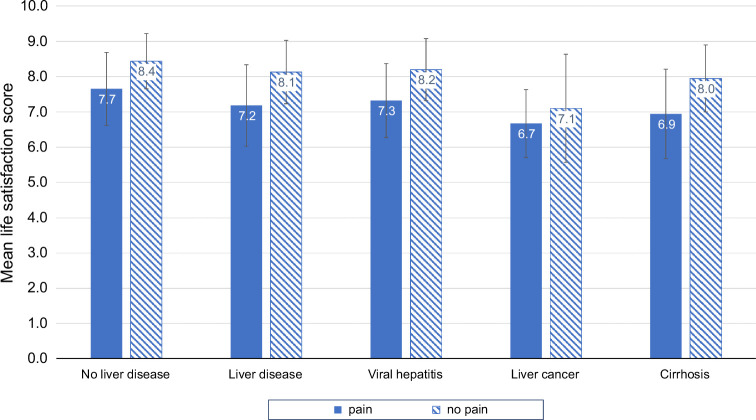
Mean life satisfaction score among patients with and without liver disease, by pain category (2021 survey), *p < 0.001.

Using the 2021 questionnaire, we sought to compare the quality of life among patients with and without pain. We found that for all subgroups, mean life satisfaction score was lower among those with pain compared to those without pain (Figure 5). Mean life satisfaction scores were higher for those without liver disease compared to those with any type of liver disease. Among those with liver disease, mean life satisfaction scores were lowest among those with pain and either liver cancer or cirrhosis.

### Opioid use in respondents with liver disease

We sought to understand patterns of opioid use among those with liver disease using responses from the 2020 NHIS. Compared to those without liver disease, a significantly greater proportion of participants with liver disease reported prescription opioid use in the preceding 12 months (29% with vs. 17% without, *p* < 0.001). Among those who reported opioid use in the past 12 months, those with liver disease were also more likely to report use in the preceding 3 months (69% vs. 59%, *p* = 0.004). Participants without liver disease were more likely to have been prescribed opioids for acute pain compared to those with liver disease (48% with vs. 61% without, *p* = 0.015), but had similar rates of use for chronic pain (65% vs. 57%, *p* = 0.13).

On multivariable logistic regression, a diagnosis of liver disease was independently associated with a nearly 50% increased odds of using opioids within the preceding 12 months, even after adjustment for age, sex, race, ethnicity, education, urbanicity, presence of pain, severity of pain, and functionally limiting pain (OR: 1.47, 95% CI: 1.12–1.92, *p* = 0.0054) (Table 3).

**TABLE 3:** Unadjusted and adjusted logistic regression predicting opioid use

Predictors of opioid use	Unadjusted OR (95% CI)	*p*	Adjusted OR (95% CI)	*p*
Liver disease				
No	Reference		Reference	
Yes	1.97 (1.61, 2.41)	<0.0001	1.47 (1.12, 1.92)	0.005
Age	0.99 (0.97, 1.01)	0.205	1.00 (0.99, 1.00)	0.041
Sex				
Female	1.11 (1.04, 1.19)	0.003	1.03 (0.90, 1.18)	0.633
Male	Reference		Reference	
Race				
Alaskan/Indian	1.55 (1.06, 2.24)	0.022	1.16 (0.60, 2.22)	0.665
Asian	0.55 (0.44, 0.68)	<0.0001	0.65 (0.41, 1.04)	0.074
Black/African	1.11 (0.99, 1.24)	0.069	0.91 (0.74, 1.11)	0.349
Other/Multiple	1.11 (0.87, 1.41)	0.423	0.98 (0.64, 1.51)	0.936
White	Reference		Reference	
Ethnicity				
Non-Hispanic	Reference		Reference	
Hispanic	0.90 (0.80, 1.02)	0.103	0.94 (0.71, 1.24)	0.641
Education				
Never attended	0.41 (0.13, 1.35)	0.145	0.69 (0.08, 6.17)	0.738
High school	Reference		Reference	
Associates degree	1.06 (0.97, 1.16)	0.171	1.24 (1.06, 1.45)	0.007
Bachelor’s degree	0.79 (0.72, 0.87)	<0.0001	1.06 (0.87, 1.28)	0.561
Postgraduate degree	0.69 (0.62, 0.78)	<0.0001	1.03 (0.83, 1.29)	0.761
Urbanicity				
Large central metropolitan	Reference		Reference	
Large fringe metropolitan	1.05 (0.95, 1.16)	0.315	1.04 (0.86, 1.27)	0.672
Medium and small metropolitan	1.18 (1.07, 1.29)	0.001	1.06 (0.89, 1.26)	0.491
Nonmetropolitan	1.33 (1.20, 1.48)	<0.0001	1.13 (0.93, 1.38)	0.221
Reporting pain				
No	Reference		Reference	
Yes	3.72 (3.46, 4.00)	<0.0001	1.64 (1.41, 1.91)	<0.0001
Severe pain				
No	Reference		Reference	
Yes	2.16 (1.97, 2.37)	<0.0001	1.73 (1.51, 1.99)	<0.0001
Functionally limiting pain				
No	Reference		Reference	
Yes	4.00 (3.55, 4.51)	<0.0001	1.92 (1.65, 2.25)	<0.0001

## DISCUSSION

In this contemporary, nationally representative sample, we found that liver disease diagnoses were strongly associated with disproportionate rates of pain and opioid use. Rates of pain among patients with liver disease were higher than in the general population, and appear to be rising more rapidly. Pain in those with liver disease was often severe—with proportions of severe pain and resulting functional limitations that were often similar to or higher than those in patients with other painful chronic conditions, such as diabetes and arthritis. Among those with liver disease, a diagnosis of liver cancer was most likely to be associated with severe pain, functional limitations due to pain, and lower life satisfaction.

Previous studies of pain in liver disease have focused on those with more advanced disease, typically those with cirrhosis, finding rates as high as 80%.^1^,^2^,^3^ In our cohort, which includes individuals with cirrhosis, as well as those without cirrhosis but with liver cancer or viral hepatitis, we found slightly lower proportions, with 43% reporting pain most or every day. Among patients with either cirrhosis or liver cancer, ~50% reported pain on most or all days, which represents a substantial proportion of the growing population of patients with liver disease. Moreover, survey respondents reported that a substantial proportion of this pain is severe and significantly impairs functional status. In all subgroups, pain was also associated with lower life satisfaction scores. This was particularly true for patients with liver cancer. While prior studies have shown high rates of pain in patients with HCC,^9^ this is one of the first to characterize pain in the population and to compare to those with chronic liver disease but without cancer.

In addition to more accurately characterizing pain among those with chronic liver disease, our study is also one of the first to compare pain in chronic liver disease with other chronic conditions, and we believe it is the first to do this in a nationally representative population. It has previously been acknowledged that pain prevalence in end-stage liver disease is similar to that in other chronic conditions.^1^ However, direct comparisons between rates of pain in different chronic conditions have been difficult to make. We were able to leverage a large cohort that included both detailed pain data as well as information on other chronic conditions; this unique data set allowed us to make these comparisons. Our study indicates that the prevalence of pain in patients with liver disease is significant, aligning with existing literature on kidney disease, diabetes, and arthritis.^10^ Notably, our findings reveal that pain reports in liver conditions such as cirrhosis are comparable to other traditionally recognized painful conditions and, therefore, we argue, should also be considered by the medical field as a “painful condition.” We hope that defining liver disease as a painful condition will stimulate future research exploring the etiology of pain in liver disease and safe and effective pain management strategies, significantly improving the quality of life for liver disease patients.

While providers have traditionally suggested that pain in chronic liver disease is most strongly associated with spontaneous bacterial peritonitis or tense ascites, our findings suggest that there are likely other causes of pain at play. Even among respondents in our liver disease cohort with the lowest rates of pain—those without cirrhosis or liver cancer (ie, those with viral hepatitis) and presumably no ascites—rates of pain and severe pain were significantly higher than the general population. In addition, rates of pain in every body location were higher among those with liver disease, not just the abdomen. These findings are consistent with previously published work suggesting widespread pain among patients with chronic liver disease^11^,^12^ and suggest that perhaps we need to rethink our traditional thinking about the etiology of pain in this population, as well as implications for its management.

In addition to ascites and spontaneous bacterial peritonitis, why might patients with liver disease experience more pain and more severe pain than those without liver disease? It is possible that patients with liver disease have more pain due to the presence of metabolic comorbidities, such as diabetes. Interestingly, in our comparison between those with liver disease and diabetes (excluding those with both conditions), we saw similar rates of pain and severe pain between the 2 groups, suggesting that the etiology of pain may be separate from diabetes. Substance use and psychiatric disorders, which are also very common in patients with chronic liver disease, may also play a role by altering pain or analgesic tolerance.^13^ Moreover, the proinflammatory state of patients with liver disease may exacerbate the sensation of pain.^12^ Pain widespreadness, which we found to be common in those with liver disease, can suggest the presence of nociplastic pain, a complex type of pain that reflects a multifaceted health picture involving emotional, cognitive, and sleep disturbances which may be less straightforward to treat than somatic pain.^11^ Indeed, there is a tendency for pain to be undertreated in this population.^14^ Medical professionals must navigate these complexities carefully to provide effective and safe pain relief.

Safely and effectively managing pain in patients with liver disease presents several unique challenges. Opioids—one of the most commonly prescribed analgesic medications—are associated with risks in the general population, including addiction, overdose, and death. In patients with liver disease, there are additional risks due to impaired hepatic metabolism, including worsening HE and adverse transplant outcomes.^15^,^16^,^17^ Despite these risks, we have previously shown that patients with liver disease—cirrhosis specifically—receive more and higher doses of opioids compared to those without.^13^,^18^ In the present study, we have again shown that patients with liver disease report higher rates of opioid use compared to the general population. While they are less likely to use opioids for acute pain, they are just as likely as the general population to use them for chronic pain, even after adjustment for severity of pain. These findings suggest that providers may be more likely to prescribe opioids to this population, possibly due to concerns about the adverse effects of other analgesic classes, such as acetaminophen and nonsteroidal anti-inflammatories. Although usage of these alternatives was not captured in this study, their underutilization in the liver disease population has been documented in other cohorts.^18^ Yet by avoiding these non-opioid alternatives, we may be predisposing an already high-risk population to additional opioid-related adverse effects.

Compared to other national pain surveillance systems, NHIS stands out as a premier source for monitoring chronic pain, offering comprehensive insights into the prevalence and patterns of pain across the US population. Despite its strengths, the NHIS relies on self-reported data which can have limitations in reliability and have a potential for recall bias. It also is mostly representative of the noninstitutionalized population so it may underrepresent the elderly population who may be under institutional care. It is important to highlight that in 2019, the NHIS implemented a redesign of their questionnaire, which could hinder the comparability of estimates obtained before and after this redesign period. However, we did avoid any data that was included from the year 2019 to mitigate this. We were limited in our ability to characterize the type or severity of liver disease (including compensation status) based on limited liver-related survey questions. However, capturing those with liver cancer and viral hepatitis allowed for the evaluation of a much broader population than typically studied. We were additionally limited in our ability to fully characterize pain, as pain was only one of many health factors assessed in this survey, and the specific pain-related questions asked have not been validated in patients with liver disease. Future prospective research in this area should incorporate symptom assessments that have been validated in patients with liver disease, to more appropriately target treatment options.^19^

In summary, in this contemporary nationally representative study using data from the NHIS from 2016 to 2021, we found that liver disease is an independent predictor of pain. This pain is often widespread, severe, and functionally limiting, prompting higher rates of opioid use in those with liver disease compared to the general population. While the relationship between liver disease–related complications, pain, and other symptoms is undoubtedly complex, our findings underscore the importance of pain management in patients with liver disease and highlight the need for tailored strategies that consider the unique challenges and risks this population faces. Perhaps clinicians should think of liver disease as a painful chronic condition—similar to how we acknowledge the close association between pain and other chronic diseases. We hope that such reframing will ensure that sufficient clinical and research resources are devoted to helping our patients adequately treat their pain and other symptoms, significantly improving their quality of life.

## Supplementary Material

SUPPLEMENTARY MATERIAL

## References

[1] Peng JK Hepgul N Higginson IJ Gao W. Symptom prevalence and quality of life of patients with end-stage liver disease: A systematic review and meta-analysis. Palliat Med. 2019;33:24–36. 30345878 10.1177/0269216318807051 PMC6291907

[2] Rogal SS Winger D Bielefeldt K Szigethy E. Pain and opioid use in chronic liver disease. Dig Dis Sci. 2013;58:2976–2985. 23512406 10.1007/s10620-013-2638-5 PMC3751995

[3] Marchesini G Bianchi G Amodio P Salerno F Merli M Panella C. Factors associated with poor health-related quality of life of patients with cirrhosis. Gastroenterology. 2001;120:170–178. 11208726 10.1053/gast.2001.21193

[4] Rikard SM Strahan AE Schmit KM Guy GP Jr. Chronic pain among adults—United States, 2019-2021. MMWR Morb Mortal Wkly Rep. 2023;72:379–385. 37053114 10.15585/mmwr.mm7215a1 PMC10121254

[5] Dennis JA Zhang Y Curtis S Brismée JM Sizer PS. Conventional and complementary health care approaches used by American adults reporting joint pain: Patterns from the National Health Interview Survey 2012. J Altern Complement Med. 2020;26:1080–1083. 32757943 10.1089/acm.2020.0237

[6] Walitt B Katz RS Bergman MJ Wolfe F. Three-quarters of persons in the US population reporting a clinical diagnosis of fibromyalgia do not satisfy fibromyalgia criteria: The 2012 National Health Interview Survey. PLoS One. 2016;11:e0157235. 27281286 10.1371/journal.pone.0157235 PMC4900652

[7] Duca LM Helmick CG Barbour KE Nahin RL Von Korff M Murphy LB. A review of potential national chronic pain surveillance systems in the United States. J Pain. 2022;23:1492–1509. 35421595 10.1016/j.jpain.2022.02.013 PMC9464678

[8] Ufere NN Lago-Hernandez C Alejandro-Soto A Walker T Li L Schoener K. Health care-related transportation insecurity is associated with adverse health outcomes among adults with chronic liver disease. Hepatol Commun. 2024;8:e0358. 38206200 10.1097/HC9.0000000000000358 PMC10786597

[9] Kaiser K Mallick R Butt Z Mulcahy MF Benson AB Cella D. Important and relevant symptoms including pain concerns in hepatocellular carcinoma (HCC): A patient interview study. Support Care Cancer. 2014;22:919–926. 24258355 10.1007/s00520-013-2039-5

[10] Aslam A Singh J Rajbhandari S. Prevalence of painful diabetic neuropathy using the self-completed Leeds Assessment of Neuropathic Symptoms and Signs Questionnaire in a population with diabetes. Can J Diabetes. 2015;39:285–295. 25935401 10.1016/j.jcjd.2014.12.007

[11] Holman A Parikh ND Zhao Z Nikirk S Clauw DJ Williams DA. Association between widespread pain and associated symptoms in patients with cirrhosis. Hepatol Commun. 2023;7:e0120. 37058114 10.1097/HC9.0000000000000120 PMC10109455

[12] Rogal SS Bielefeldt K Wasan AD Szigethy E Lotrich F DiMartini AF. Fibromyalgia symptoms and cirrhosis. Dig Dis Sci. 2015;60:1482–1489. 25433921 10.1007/s10620-014-3453-3 PMC4688457

[13] Rogal SS Winger D Bielefeldt K Rollman BL Szigethy E. Healthcare utilization in chronic liver disease: The importance of pain and prescription opioid use. Liver Int. 2013;33:1497–1503. 23758842 10.1111/liv.12215 PMC3795935

[14] Rubin JB Lai JC Shui AM Hohmann SF Auerbach A. Patterns of inpatient opioid use and related adverse events among patients with cirrhosis: A propensity-matched analysis. Hepatol Commun. 2021;5:1081–1094. 34141991 10.1002/hep4.1694 PMC8183179

[15] Rogal S Dew MA DiMartini A. High-dose opioid use and liver transplantation: An underestimated problem? Liver Transpl. 2017;23:285–287. 28133898 10.1002/lt.24731

[16] Acharya C Betrapally NS Gillevet PM Sterling RK Akbarali H White MB. Chronic opioid use is associated with altered gut microbiota and predicts readmissions in patients with cirrhosis. Aliment Pharmacol Ther. 2017;45:319–331. 27868217 10.1111/apt.13858

[17] Moon AM Jiang Y Rogal SS Tapper EB Lieber SR Barritt AS IV. Opioid prescriptions are associated with hepatic encephalopathy in a national cohort of patients with compensated cirrhosis. Aliment Pharmacol Ther. 2020;51:652–660. 31960985 10.1111/apt.15639 PMC7047528

[18] Rubin JB Lai JC Shui AM Hohmann SF Auerbach A. Cirrhosis inpatients receive more opioids and fewer nonopioid analgesics than patients without cirrhosis. J Clin Gastroenterol. 2023;57:48–56. 34653064 10.1097/MCG.0000000000001624 PMC9008074

[19] Patel AA Tapper EB Kanwal F Woodrell CD Hansen L Lai JC. Targets and study design for symptom-focused trials aimed at patients with cirrhosis: An expert consensus. Hepatol Commun. 2023;7:e0135. 37267219 10.1097/HC9.0000000000000135 PMC10241502

