# Supplementary material for: Pain in chronic liver disease compared to other chronic conditions: Results from a contemporary nationally representative cohort study
Source: Hepatol Commun. 2024 Dec 11;9(1):e0605. doi: 10.1097/HC9.0000000000000605 (PMC11637743; doi:10.1097/HC9.0000000000000605)
Supplement: SUPPLEMENTARY MATERIAL [file hc9-9-e0605-s001.docx]

**Supplemental Table 1**: Disease Definitions by NHIS Survey Questions

| Condition | Survey Question |
| --- | --- |
| Liver Disease | |
| Viral Hepatitis | “Have you ever been told by a doctor or other health care professional that you had had hepatitis” |
| Other Liver Disease/Cirrhosis | “Have you ever been told by a doctor or other health care professional that you had had cirrhosis/liver condition” |
| Liver Cancer | “Liver cancer mentioned” |
| Diabetes | “Have you ever been told by a doctor or other health care professional that you had diabetes” |
| Arthritis | “Have you ever been told by a doctor or other health care professional that you had arthritis” |
| Chronic Kidney Disease | “Have you ever been told by a doctor or other health care professional that you had weak/failing kidneys” |

**Supplemental Table 2**: Pain, Opioid Use, Life Satisfaction Definitions by NHIS Survey Questions

| Pain Category | Survey Question |
| --- | --- |
| Rates of Pain | “In the past three months, how often did you have pain? Would you say never, some days, most days, or every day?" |
| Severity of pain | “Thinking about the last time you had pain, how much pain did you have? Would you say a little, a lot, or somewhere in between?” |
| Functional Limitations by Pain | “Over the past three months, how often did your pain limit your life or work activities? Would you say never, some days, most days, or every day?” |
| Opioid Use | “During the past 12 months, have you taken any opioid pain relievers prescribed by a doctor, dentist, or other health professional?” |
|  | “During the past 3 months, have you taken any opioid pain relievers prescribed by a doctor, dentist, or other health professional?” |
|  | “During the past 3 months, did you take a prescription opioid to treat short term or acute pain, such as pain due to a broken bone or muscle sprain, pain from dental work, or pain following surgery?” |
|  | “During the past 3 months, did you take a prescription opioid to treat long term or chronic pain, such as low back pain or neck pain, frequent headaches or migraines, or joint pain or arthritis?” |
|  | “During the past 3 months, how often did you take a prescription opioid? Would you say some days, most days, or every day?” |
| Pain location | “Over the past three months, how much have you been bothered by…back pain, pain in hands, pain in hips, migraine, abdominal pain, toothache/jaw pain…Would you say not at all, a little, a lot, or somewhere in between?” |
| Quality of life / life satisfaction | “Using a scale of 0 to 10, where 0 means "very dissatisfied" and 10 means "very satisfied", how do you feel about your life as a whole these days?” |

**Supplemental Table 3.** Adjusted logistic regression predicting pain, including adjustment for comorbidities.


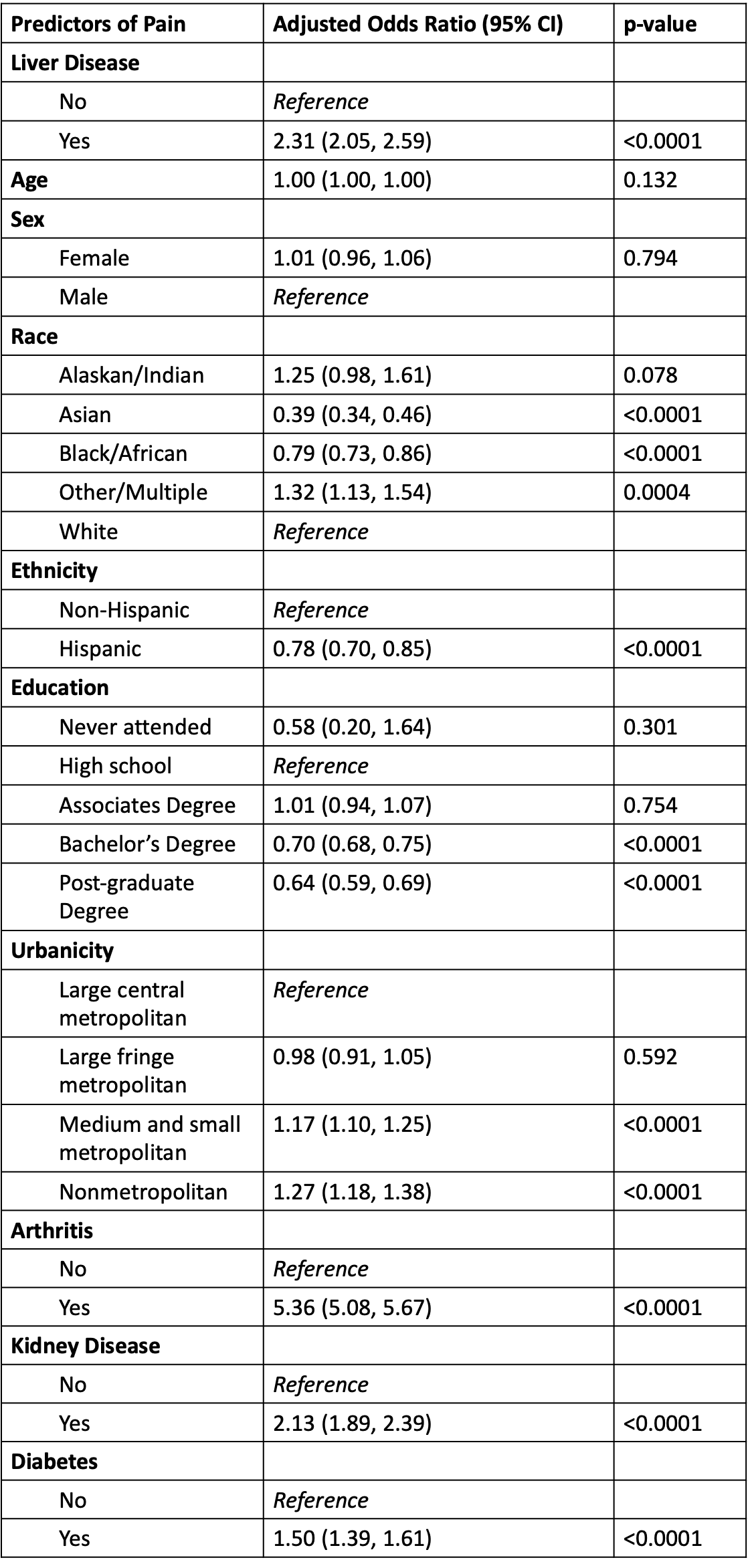


**Supplemental Table 4.** Odds of having pain among those with liver disease, by liver disease type (n = 5267). Adjusted model was adjusted for age, sex, race, ethnicity, education, and urbanicity.
